# Complicated appendicitis, acute pancreatitis, pleural effusion, and sinus bradycardia in a COVID‐19 patient

**DOI:** 10.1002/ccr3.7077

**Published:** 2023-03-08

**Authors:** Danial Hatami, Seyed Mohamamd Amin Alavi

**Affiliations:** ^1^ Faculty of Medicine Ahvaz Jundishapur University of Medical Sciences Ahvaz Iran

**Keywords:** appendicitis, bradycardia, COVID‐19, pancreatitis, SARS‐CoV‐2

## Abstract

This study shows that complicated appendicitis and acute pancreatitis could occur during a COVID‐19 infection, since the same gastrointestinal manifestations are notable in all aforementioned diseases. Sinus bradycardia is a side effect of remdesivir. Both COVID‐19 infection and remdesivir therapy can elevate liver transaminases.

## 1 INTRODUCTION

The Wuhan Municipal Health Commission reported the first cluster case of pneumonia in Wuhan, Hubei Province, China, on the last day of 2019. Chinese authorities detected a novel coronavirus variant, which was isolated a week later and named SARS‐CoV‐2.^1^ The World Health Organization (WHO) officially started a public health emergency on January 30, 2020, regarding the onset of COVID‐19 disease.^2^ As of November 11, 2022, WHO reported over 630 million confirmed cases worldwide, resulting in over 6.5 million deaths.^3^ Fever, coughing, shortness of breath, nausea or vomiting, and diarrhea are frequent symptoms that may appear 2–14 days following viral exposure.^4^ Diarrhea and abdominal pain are the most often reported gastrointestinal symptoms related to pancreatic involvement in COVID‐19 patients.^5^ Classic symptoms of appendicitis are periumbilical pain and low‐grade fever,^6^ which can also be seen in COVID‐19.

Previous studies have indicated that COVID‐19 could result in decreased uncomplicated appendicitis and similar or an increased number of complicated appendicitis in non‐pandemic versus pandemic time periods.^7^, ^8^ SARS‐CoV‐2 uses the angiotensin‐converting enzyme (ACE2) to infect cells. This receptor can be found not only in the lungs but also in other organs, including the gut and pancreas, making them a potential target for SARS‐CoV‐2.^9^

Herein, we report a case of appendicitis and pancreatitis in a patient with COVID‐19 infection.

## 2 CASE PRESENTATION

The Patient was a 29‐year‐old man without any previous medical history. He was vaccinated with two doses of the Sinopharm vaccine 6 months before these events. He was a social drinker, and he smoked five cigarettes a day. The body mass index (BMI) was 24.66 $\frac{\mathrm{kg}}{m^{2}}$.

The initial symptoms were sore throat, rhinorrhea, and fever, which started 2 days before admission. The SARS‐CoV‐2 real‐time reverse transcriptase‐polymerase chain reaction (real‐time RT‐PCR) test was reported to be positive a day after the initial symptoms. He complained of abdominal cramps a day before admission. Previous symptoms abated and epigastric pain, nausea, and vomiting started on the morning of admission. The pain shifted to the right lower quadrant region of the abdomen. The Alvarado score at this time was 8 (Only leukocytosis >10,000 was not seen in the patient's lab test). An ultrasound investigation was carried out for the patient, which showed target‐shaped appendix with 9 mm diameter. The vital signs on arrival time were as follows: blood pressure:120/80 mmHg, pulse rate 83, respiratory rate 23, and body temperature of 38°C. Moreover, O2 saturation was 98% breathing ambient air at the time of admission.

**FIGURE 1:**
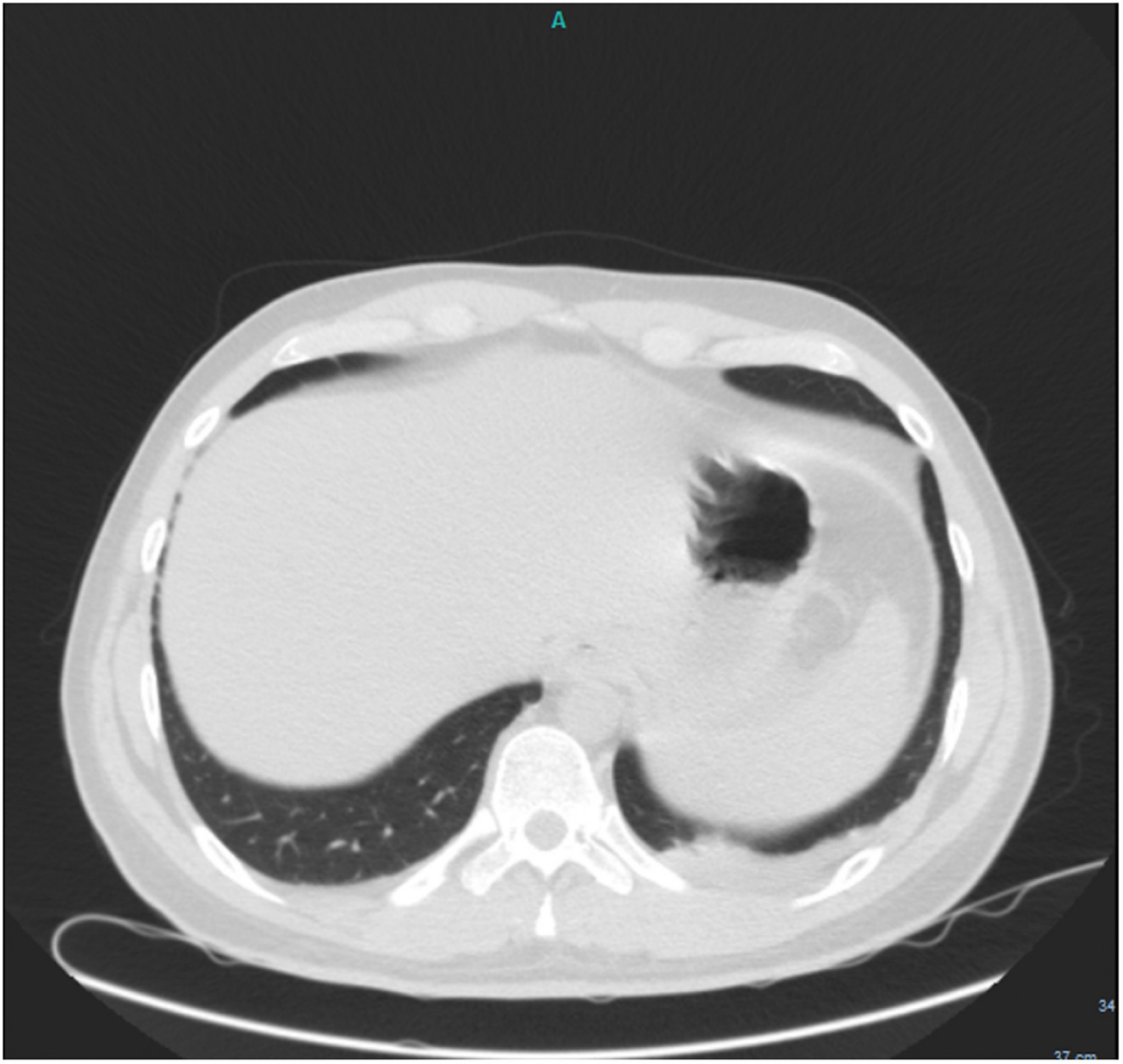
Chest computed tomography scan, pulmonary window, axial, showing left‐side pleural effusion.

**FIGURE 2:**
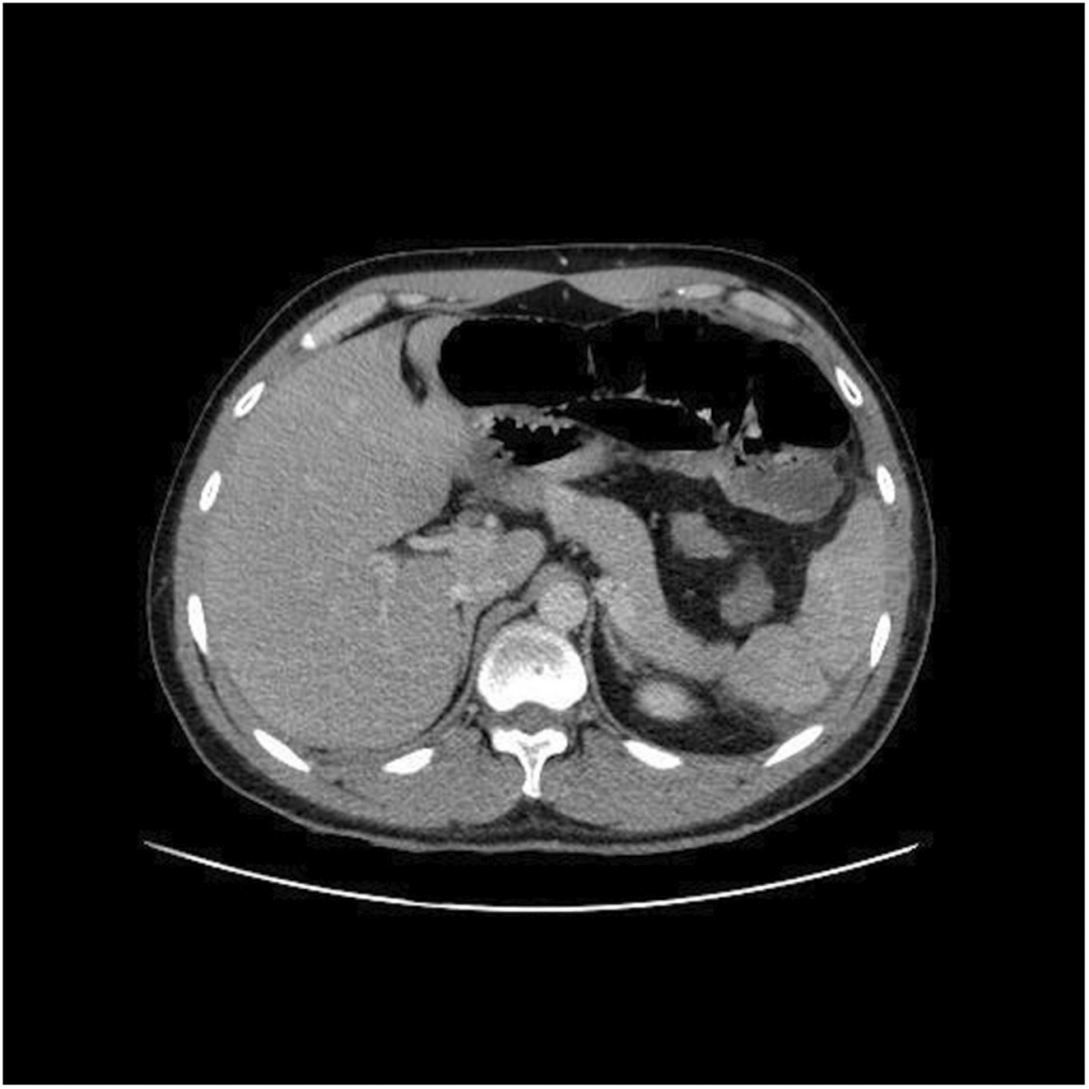
Normal abdominal computed tomography scan with contrast, portal phase, without abnormal pancreatic radiologic findings.

Ceftriaxone 1 gr and metronidazole 500 mg were ordered intravenously at admission and continued for 4 days. He went through a laparoscopic appendectomy on the same day, in which phlegmon was reported. Abdominal cramps and diarrhea started the next morning. He started a liquid diet 1 day after surgery, but the pain worsened. Another ultrasound was carried out on the fourth day of hospitalization, which was normal. Chest high‐resolution computed tomography (Chest HRCT) was carried out on the fifth day of hospitalization, in which there was no grand glass opacity or lung involvement; however, left‐side pleural effusion was observed (Figure 1). An abdominal CT scan was ordered on the same day, which was normal (Figure 2). The amylase level was reported 317 U/L on the 4th day of hospitalization and increased continuously (all laboratory findings are presented in Table 1). Acute pancreatitis was diagnosed based on Atlanta criteria, and the patient was transferred to the intensive care unit (ICU). The diet changed to nil per os (NPO), and fluid resuscitation started for the patient. Remdesivir 100 mg daily (with a loading dose of 200 mg) and dexamethasone 8 mg three times daily were ordered on the sixth day of hospitalization. On the seventh day of hospitalization, after the second dose of remdesivir, bradycardia was seen during the cardiac monitoring. The heart rate was 35–40 beats per minute (BPM) during awakening and 25 during sleep, for which remdesivir was discontinued. Heparin 1000 unit/hour was ordered intravenously due to D‐dimer elevation on the seventh day of hospitalization. The patient recovered gradually and was discharged 11 days after admission. As well, after performing several evaluations, the patient was found in normal condition after discharge. Magnetic resonance cholangiopancreatography was performed after discharge and did not evidence any abnormality.

**TABLE 1:** Laboratory findings

Laboratory parameter	A D	H D 1	H D 2	H D 3	H D 4	H D 5	H D 6	H D 7	H D 8	H D 9	H D 10
Leukocyte ×1000	7.4	ND	ND	7.6	7.1	6.94	6.7	8.17	7.4	8.2	11.22
Lymphocyte (%)	17	ND	ND	12	10	15	10	19	21	30	15
Neutrophils (%)	78	ND	ND	83	80	79	86	76	74	65	81
Platelets ×1000	150	ND	ND	188	192	231	247	291	385	363	385
Hemoglobin (gr/dL)	14.7	ND	ND	13.4	13.2	12.5	12.2	11.5	12.5	12.5	13.4
ESR (mm/hr)	9	ND	ND	ND	ND	60	ND	47	27	24	17
CRP (mg/L)	5.5	ND	ND	ND	ND	Trace	54.4	50	49	22.8	46
BUN (mg/dL)	9	ND	ND	7	7	9	9	12	13	12	11
Cr (mg/dL)	1.3	ND	ND	1.1	1.04	0.94	0.83	0.78	0.9	0.9	1.02
Na (mEq/L)	137	ND	ND	137	138	143	140	139	139	141	137
K (mEq/dL)	4.3	ND	ND	3.8	4	4.9	4.7	4.9	4.2	4.1	4
Ca (mEq/dL)	ND	ND	ND	ND	ND	8.6	ND	ND	ND	ND	ND
P (mEq/dL)	ND	ND	ND	ND	ND	2.7	ND	ND	ND	ND	ND
AST (IU/L)	30	22	ND	ND	11	15	ND	ND	ND	81	ND
ALT (IU/L)	55	36	ND	ND	19	18	ND	ND	ND	90	ND
ALP (IU/L)	154	125	ND	ND	132	123	ND	ND	ND	115	ND
Bili T (mg/dL)	1.1	1.3	ND	ND	0.76	0.69	ND	ND	ND	0.7	ND
Bili D (mg/dL)	0.23	0.3	ND	ND	0.19	0.16	ND	ND	ND	0.2	ND
LDH (IU/L)	ND	ND	ND	ND	ND	402	ND	ND	ND	ND	ND
BS (mg/dL)	120	ND	ND	ND	96	ND	150	110	94	87	94
Albumin (g/L)	ND	ND	ND	ND	ND	4.5	ND	ND	ND	ND	ND
ABG (PH)	7.39	ND	ND	ND	7.35	ND	7.36	ND	ND	ND	ND
ABG (PCO2)	44	ND	ND	ND	41	ND	33	ND	ND	ND	ND
ABG (PaO2)	27	ND	ND	ND	24	ND	30	ND	ND	ND	ND
ABG (HCO3)	26.6	ND	ND	ND	22.6	ND	18.6	ND	ND	ND	ND
D‐Dimer (ng/mL)	ND	ND	ND	ND	ND	ND	16,960	12,320	ND	8530	9320
Amylase (U/L)	121	137	ND	ND	317	523	601	821	688	ND	1072
Lipase (U/L)	80	55	ND	ND	224	201	377	540	600	ND	913
PT (Sec)	14.7	ND	ND	ND	ND	17	16.5	16.1	15.7	15.5	16.4
PTT (Sec)	35	ND	ND	ND	ND	34.7	38	35	34.1	36.9	36
INR	1.18	ND	ND	ND	ND	1.38	1.3	1.4	1.27	1.2	1.3
S/E (RBC)	ND	ND	ND	Many	ND	ND	ND	Many	ND	ND	ND
S/E (WBC)	ND	ND	ND	2–4	ND	ND	ND	6–8	ND	ND	ND
S/E (ova of parasite)	ND	ND	ND	0	ND	ND	ND	0	ND	ND	ND
S/E (Protozoa)	ND	ND	ND	0	ND	ND	ND	0	ND	ND	ND
S/C	ND	ND	ND	0	ND	ND	ND	0	ND	ND	ND
Fecal calprotectin	ND	ND	ND	ND	ND	ND	ND	Neg	ND	ND	ND

Abbreviations: ABG, arterial blood gas test; AD, admission day; ALP, alkaline‐phosphatase; ALT, alanine aminotransferase; AST, aspartate aminotransferase; Bili D, bilirubin direct; Bili T, bilirubin total; BS, blood sugar; BUN, blood urea nitrogen; Ca, calcium; Cr, creatinine; CRP, C‐reactive protein; ESR, erythrocyte sedimentation rate; HD, hospitalization day; INR, international normalized ratio; K, potassium; LDH, lactate‐dehydrogenase; Na, sodium; ND, not determined; Neg, negative; P, phosphorus; PT, prothrombin time; PTT, partial thromboplastin time; RBC, red blood cell; S/C, stool culture; S/E, stool examination test; WBC, white blood cell.

## 3 DISCUSSION

Simple appendicitis (e.g., early or uncomplicated appendicitis) and complicated appendicitis (e.g., gangrenous appendicitis and appendiceal phlegmon or abscess) are the two major subcategories of acute appendicitis.^10^ As mentioned earlier, the rate of complicated appendicitis increased during the COVID‐19 era.^8^ Despite the early referral for the surgery, a phlegmon was formed, and complicated appendicitis occurred.

Pleural effusion was reported in this patient. The presence of pleural effusion can be used as a prognostic indicator to determine the probability that COVID‐19 patients are highly probable to experience worse outcomes.^11^ A previous study by Peycru et al. reported rare cases of pleural effusion after laparoscopic appendectomy; however, all of them were right‐sided.^12^ Pleural effusion is also a frequent chest finding in the presence of acute pancreatitis, in which the volume of pleural effusion is a valid radiological biomarker for predicting the severity and clinical prognosis of acute pancreatitis.^13^

Acute pancreatitis is defined by inflammation of the exocrine pancreas and acinar cell damage, which leads to intrapancreatic protease activation and acute pancreatitis.^14^ Two main etiologies of acute pancreatitis are gallstone and alcohol consumption.^15^ Based on a review by Jablonska et al., SARS‐CoV‐2 may be a new etiology for acute pancreatitis.^16^ The diagnosis of acute pancreatitis requires the presence of two of three criteria: (1) upper abdominal pain, (2) serum amylase or lipase (or both) more than three times the upper limit of normal, and (3) imaging findings (ultrasound, CT scan, magnetic resonance imaging).^15^

Amylase level was slightly elevated in this patient at admission, and both amylase and lipase were three times above normal values on the fourth day of hospitalization. Pancreatic enzyme elevation is frequent among patients suffering from COVID‐19 and related to poor prognosis.^9^ The criteria did not manifest themselves during the admission of the patient. However, as the abdominal pain worsened and the serum amylase increased threefold higher than what is normal, acute pancreatitis was diagnosed.

Sinus bradycardia occurred after the second dose of remdesivir was given to the patient, however, within 24 h after drug discontinuation the symptoms abated. Previous studies had also indicated the occurrence of sinus bradycardia as a side effect of remdesivir.^17^, ^18^, ^19^

D‐dimer level elevated on the sixth day of hospitalization. A systematic review and meta‐analysis by Paliogiannis et al. revealed that patients with severe COVID‐19 have considerably greater blood D‐dimer concentrations than those with less severe forms.^20^ It is suggested to conduct CT pulmonary angiography for COVID‐19 patients with elevated D‐dimer lever^21^; however, as the patient did not consent to CT pulmonary angiography, the procedure could not be carried out. Another laboratory marker which was elevated in the patient was c‐reactive protein (CRP). Based on a previous study by Malik et al., elevated CRP level is a biomarker that suggests the severity of COVID‐19.^22^ CRP > 150 mg/L is considered a gold standard 48 hours after acute pancreatitis onset, yet this was lower in the patient.^23^

Liver enzymes were slightly elevated at the time of admission but reached normal levels on the first day of hospitalization. A previous study by Wijarnpreecha et al. stated that liver enzymes were elevated in one‐fourth of COVID‐19 patients and were associated with disease severity.^24^ On the eighth day of hospitalization, liver transaminases level were elevated again. A study by Van Laar et al. stated that liver transaminases were elevated in 35% of the patients receiving remdesivir, which should not be an absolute contraindication if they are regularly monitored.^25^

## 4 CONCLUSION

Gastrointestinal manifestation of COVID‐19 is perceived to be a sign of appendicitis, which could alert the attending physician to pay further attention to other diseases during COVID‐19 infection. COVID‐19 can elevate pancreatic enzymes with or without pancreatic injury. Clinicians should be careful about pancreatitis in COVID‐19 patients. Liver transaminases could be elevated in SARS‐CoV‐2‐positive patients and those who receive remdesivir, thus indicating closer monitoring of patients for liver injury. Remdesivir also can cause sinus bradycardia, subsequently, the drug should be discontinued as soon as possible.

## AUTHOR CONTRIBUTIONS

**Danial Hatami:** Writing – original draft. **Seyed Mohamamd Amin Alavi:** Writing – original draft; writing – review and editing.

## FUNDING INFORMATION

No sources of funding were declared for this study.

## CONFLICT OF INTEREST STATEMENT

The authors have no conflict of interest to declare.

## ETHICAL APPROVAL STATEMENT

There was no need for the ethics committee's permission for case reports in the institution where the research was carried out.

## CONSENT

Written informed consent for publication of the case report has been signed by the patient and is available upon request from the editors.

## Data Availability

The data that support the finding of this study are available upon request from the corresponding author.
