#  Correction to “Complicated appendicitis, acute pancreatitis, pleural effusion, and sinus bradycardia in a COVID‐19 patient”

**DOI:** 10.1002/ccr3.7493

**Published:** 2023-09-25

Correction to “Complicated appendicitis, acute pancreatitis, pleural effusion, and sinus bradycardia in a COVID‐19 patient

Hatami, D, Alavi, SMA. Complicated appendicitis, acute pancreatitis, pleural effusion, and sinus bradycardia in a COVID‐19 patient. Clin Case Rep. 2023; 11:e7077. doi:10.1002/ccr3.7077

In the article entitled “Complicated appendicitis, acute pancreatitis, pleural effusion, and sinus bradycardia in a COVID‐19 patient” that was previously published in Volume 11 Issue 3 of Clinical Case Reports, the 2nd authors' name was misspelt.

The correct name should be: “Seyed Mohammad Amin Alavi”.

We apologize for this error.

